# A practical tool for assessing ecosystem services enhancement and degradation associated with invasive alien species

**DOI:** 10.1002/ece3.5020

**Published:** 2019-03-27

**Authors:** Rocio Martinez‐Cillero, Simon Willcock, Alvaro Perez‐Diaz, Emma Joslin, Philippine Vergeer, Kelvin S.‐H. Peh

**Affiliations:** ^1^ School of Biological Sciences University of Southampton Southampton UK; ^2^ Wageningen University and Research Centre Wageningen The Netherlands; ^3^ Centre for Environment and Sustainability University of Surrey Guildford UK; ^4^ School of Natural Sciences Bangor University Bangor UK; ^5^ Electronics and Computer Science University of Southampton Southampton UK; ^6^ Faculty of Engineering and the Environment University of Southampton Southampton UK; ^7^ Conservation Science Group, Department of Zoology University of Cambridge Cambridge UK

**Keywords:** alternative management, expert judgment, Great Britain, non‐native, novel approach

## Abstract

Current approaches for assessing the effects of invasive alien species (IAS) are biased toward the negative effects of these species, resulting in an incomplete picture of their real effects. This can result in an inefficient IAS management. We address this issue by describing the INvasive Species Effects Assessment Tool (INSEAT) that enables expert elicitation for rapidly assessing the ecological consequences of IAS using the ecosystem services (ES) framework. INSEAT scores the ecosystem service “gains and losses” using a scale that accounted for the magnitude and the reversibility of its effects. We tested INSEAT on 18 IAS in Great Britain. Here, we highlighted four case studies: *Harmonia axyridis* (Harlequin ladybird), *Astacus leptodactylus* (Turkish crayfish), *Pacifastacus leniusculus* (Signal crayfish) and *Impatiens glandulifera *(Himalayan balsam). The results demonstrated that a collation of different experts’ opinions using INSEAT could yield valuable information on the invasive aliens’ ecological and social effects. The users can identify certain IAS as ES providers and the trade‐offs between the ES provision and loss associated with them. This practical tool can be useful for evidence‐based policy and management decisions that consider the potential role of invasive species in delivering human well‐being.

## 1 INTRODUCTION

Invasive alien species (IAS) are human‐mediated introduced species that sustain self‐replacing populations and have the potential to spread over long distances, producing reproductive offspring normally in large numbers (Richardson & Pyšek, 2008). These aliens are considered a threat to human health and economy (Simberloff, 2000), as well as one of the main causes of native species extinction (Convention on Biological Diversity, 2008; Withgott & Brennan, 2009; Wittenberg & Cock, 2001). Many have, however, questioned the direct causality between IAS dominance and native species decline in degraded systems (Didham, Tylianakis, Hutchison, Ewers, & Gemmell, 2005; Gurevitch & Padilla, 2004; Schlaepfer, Sax, & Olden, 2012; Slobodkin, 2001): some IAS are perceived as “passengers,” rather than the “drivers,” of the ecological change primarily caused by habitat modification (Byers, 2002; Corbin & D'Antonio, 2004; MacDougall & Turkington, 2005; Seabloom, Harpole, Reichman, & Tilman, 2003). Furthermore, our understanding of the socio‐economic and environmental effects of IAS could potentially be biased as a result of over‐reporting of their negative effects (Bonanno, 2016; Davis, 2009; Levine et al., 2003; McMahon, Fukami, & Cadotte, 2006; Schlaepfer, Sax, & Olden, 2011; Schlaepfer et al., 2012). In fact, there are relatively few empirical studies that present information about the benefits provided by IAS, although the focus on this literature has been increasing in the last years (Kull et al., 2011; Shackleton et al., 2007; Tassin & Kull, 2015). The so‐called “conflict species” can be highly regarded for the benefits they provide. But they can also be considered as a serious environmental threat from a management perspective.

Many risk and impact assessments have been developed to prioritize IAS control and management, with a focus on the negative environmental impacts and economic damages (Roy et al., 2014). Prevention has been increasingly recognized as the most cost‐effective strategy to ensure pristine ecosystems remaining free of IAS (Genovesi & Monaco, 2013; Meyerson & Mooney, 2007), even though it is not foolproof (Chornesky et al., 2005). IAS control and eradication are often advocated as consequent management operations and require huge financial resources (Boonman‐Berson, Turnhout, & van Tatenhove, 2014; Ewel & Putz, 2004). Yet, high rates of species invasions are projected to increase in the future. Suggestions have been proposed toward building or maintaining ecosystem resilience and services, rather than restoring IAS‐free ecosystems that may be futile (Lin & Petersen, 2013; Pyšek & Richardson, 2010). Although this approach is controversial due to the importance of the evolutionary context in species interactions (Richardson & Ricciardi, 2003) and the unpredictability of some negative consequences of invasions, there is nevertheless a pragmatic need for management alternatives to IAS removal (Hulme, Pyšek, Nentwig, & Vilà, 2009; McMahon et al., 2006).

Ecosystem services (ES) are the processes, functions or ecological characteristics through which ecosystems sustain and fulfill human life, either *directly* (e.g., provision of food) or *indirectly* (e.g., pollination) (Costanza et al., 2017; Daily, 1997). IAS may cause changes in these services by altering the ecosystems (Peh et al., 2015; Vilà et al., 2010; Vilà & Hulme, 2017). Therefore tools, such as risk‐assessment schemes, that help to evaluate such impacts and aid for the prioritization and management of IAS are essential. Roy et al., (2018) identified 14 minimum attributes a risk‐assessment scheme should include, of which two are related to human well‐being: “Assessment of impact on ecosystem services” and “Assessment of socio‐economic impacts.” These attributes were also two of the most notable gaps in our knowledge required for completing risk assessments.

However, IAS ES impact assessments are always challenging and require substantial resources for three reasons: first, ES are governed by complex interactions that make them difficult to measure over space and time; second, long‐term, large‐scale data often do not exist (Eviner, Garbach, Baty, & Hoskinson, 2012; Kremen, 2005); and last, current measures of many ES are still crude (Bennett, Peterson, & Gordon, 2009; Naidoo et al., 2008). Yet, new standards to evaluate IAS effects on human well‐being have been developed (Çinar, Arianoutsou, Zenetos, & Golani, 2014; Dickie et al., 2014; McLaughlan, Gallardo, & Aldridge, 2014; Pejchar & Mooney, 2009). An important example is the Socio‐Economic Impact Classification of Alien Taxa (SEICAT; Bacher et al., 2018) that evaluates the impacts on human welfares using changes in human activities as metric; a sister‐scheme of the Environmental Impact Classification of Alien Taxa (EICAT) which is officially adopted by IUCN. This scheme has been formulated under the assumption that IAS are drivers of the change, and purposely do not consider their positive impacts.

Here we describe the INvasive Species Effects Assessment Tool (INSEAT), a new approach that contributes to the current scenario of IAS assessment in several aspects. INSEAT significantly differs from previous attempts as it considers both positive and negative impacts of IAS on ES, with the objective to obtain a fair and informed evaluation. INSEAT uses the ES framework, commonly classified into provisioning, regulating and cultural services. This differs from SEICAT which uses the constituents of human well‐being; and EICAT, which defines its own categories of environmental impacts. The employment of the ES framework in INSEAT would aid the interpretation of the results, as it is a well‐known concept widely accepted by the conservation practitioners. Furthermore, INSEAT can provide insights on knowledge gaps within the expert community.

This practical tool, however, would not yet address complexities such as discerning effects that are temporally or spatially scale‐dependent, or accounting for biological factors such as lag‐times, dispersal, interactive effects, and environmental context. Nevertheless, INSEAT can yield valuable information for IAS managers by enabling them to (a) evaluate rapidly experts’ opinions on how IAS affects ES delivery, including positive IAS effects; (b) gather knowledge and information to enable exploration of alternative management options; (c) produce simple, graphical representation of synergies and trade‐offs among the effects of IAS; and (d) assess the management effort required to eradicate an alien species. This would make IAS management more efficient and diverse, in terms of exploring management potential that is overlooked under current methodologies. Information obtained by using INSEAT can then be fed into an integrated approach which, among other activities, involves seeking stakeholder opinions on the way forward (Cook & Proctor, 2007; Liu, Proctor, & Cook, 2010).

In this study, we piloted INSEAT to assess the effects of 18 well‐known IAS in Great Britain (GB) on ecosystem service provision. However, due to space constraint, we described only four case studies here: *Harmonia axyridis* (Harlequin ladybird), *Astacus leptodactylus* (Turkish crayfish), *Pacifastacus leniusculus* (Signal crayfish) and *Impatiens glandulifera *(Himalayan balsam). The feedback from the experts then led to a further refinement of the tool which includes an improved impact scale definition; an assessment of uncertainty on the experts’ responses; and a request of supporting information from the experts.

## 2 METHODS

A concise, yet informative, ES classification scheme is essential for IAS managers to understand the different types of ES. We built an integrated ES classification scheme (Appendix 1) based on three widely accepted ES classifications from the Millennium Ecosystem Assessment (Millennium Ecosystem Assessment, 2005), the UK National Ecosystem Assessment (UK NEA; Mace et al., 2011) and The Economics of Ecosystem and Biodiversity (TEEB, 2016). We excluded supporting services in our ES classification scheme to avoid double‐counting since all the other services are underpinned by them (Haines‐Young & Potschin, 2012).

**Figure 1:**
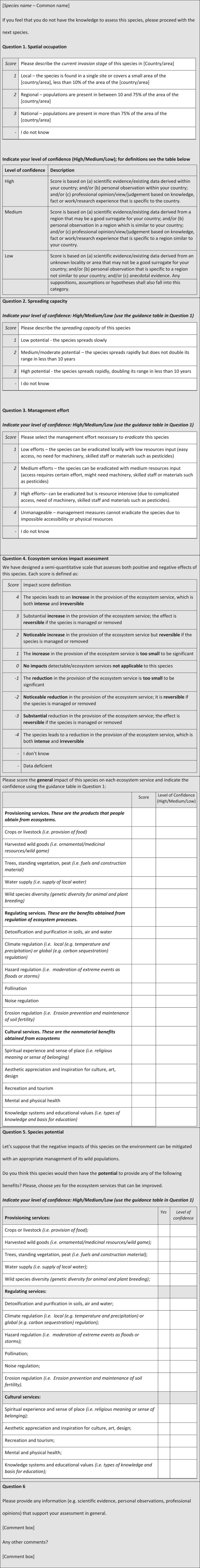
INvasive Species Effects Assessment Tool (INSEAT). Assessment form—questions and scoring system (final version). The pilot assessment form, as well as the changes implemented after respondents and reviewers’ feedback, can be found in Appendix 2

Assessing IAS effects on ES requires a qualitative and broad evaluation (Roy et al., 2014). INSEAT is designed to be completed by experts on a particular IAS by scoring its effect on a range of ES from our ES classification scheme (although other ES classifications could also be used). We created an integrated assessment proforma (Figure 1) that included questions designed to assess (a) the strength and direction of IAS effects on ES provision; (b) IAS potential to provide ES; and (c) the management effort required to eradicate the alien species.

### 2.1 Using experts’ opinions

The INSEAT protocol relies on expert judgment, which is often sought when there is scientific uncertainty or when data are absent or insufficient (Hemming, Burgman, Hanea, McBride, & Wintle, 2018). However, experts’ reliability can be compromised, as experts are prone to biases and heuristics. Hence, numerous expert elicitation techniques have been developed (Cooke, 1991; O'Hagan et al., 2006; Sutherland & Burgman, 2015). In general, experts must be tested with their estimates validated with independent evidence, in order to improve their accuracy; and independent opinions should be sought. However, expert elicitation remains largely informal and nontransparent. To improve the accuracy of expert judgment as well as the transparency of the results, Hemming et al. (2018) published a structured elicitation protocol called IDEA (Investigate, Discuss, Estimate and Aggregate). This protocol allows the experts to answer the questionnaire individually while providing reasons for their judgments; and modify their responses discreetly after reviewing the answers from other anonymous respondents.

INvasive Species Effects Assessment Tool, however, does not follow all the steps prescribed by IDEA as it does not seek to establish a definite rational consensus on IAS management. Instead, it is designed as a rapid screening tool for assessing the divergences in the opinions from a large number of IAS experts. INSEAT allows gathering of information about the sources of knowledge that these experts used (see Question 6 in Figure 1), so that the users can critically review their responses. The tool also seeks to open debate on alternative management options, which can be achieved only if a large number of experts complete the survey. By having short response times, INSEAT has the possibility to gather a high amount of responses.

A number of measures have been taken to minimize bias and improve the level of confidence: First, INSEAT stresses that the respondent should be an expert in the IAS of interest. Second, the respondents should be selected carefully—for example, we focused only on the IAS experts from Great Britain when piloting the tool, since IAS effects are mostly context dependent (Pyšek & Richardson, 2010; Vilà et al., 2011). Third, the language used in the questionnaire has been tested during the pilot phase and improved, to avoid language‐based uncertainties. Fourth, the experts are asked to gauge their level of confidence in their responses (this was added on to the final version of INSEAT after piloting). Finally, the experts are asked to provide evidence to support their answers in order to weight their opinions (this was added on to the final version of INSEAT after piloting).

### 2.2 Assessing strength and direction of IAS effects

Semiquantitative Likert scales are used to rank environmental and socio‐economic impacts, following other assessments such as the Generic Impact Scoring System (Nentwig, Bacher, Pyšek, Vilà, & Kumschick, 2016). Each scale level is well‐defined to avoid ambiguities and also to make categories and taxa comparable. The scale ranges from −4 to 4, each level combining the *strength* (“no effect,” “too small,” “noticeable,” “substantial,” and “intense”) and the *reversibility *of the impact if the species is removed (“reversible” or “irreversible”). We consider that only “intense” effects can be irreversible, as for less extreme impacts the ecosystems would naturally recover to their original state.

We used the variability of agreement among the respondents as a measure of robustness in the knowledge of a species in terms of its impact on a particular ES. Low agreement, inferred by a high variability in the scoring, helps to identify knowledge gaps about the effect of that species.

**Figure 2:**
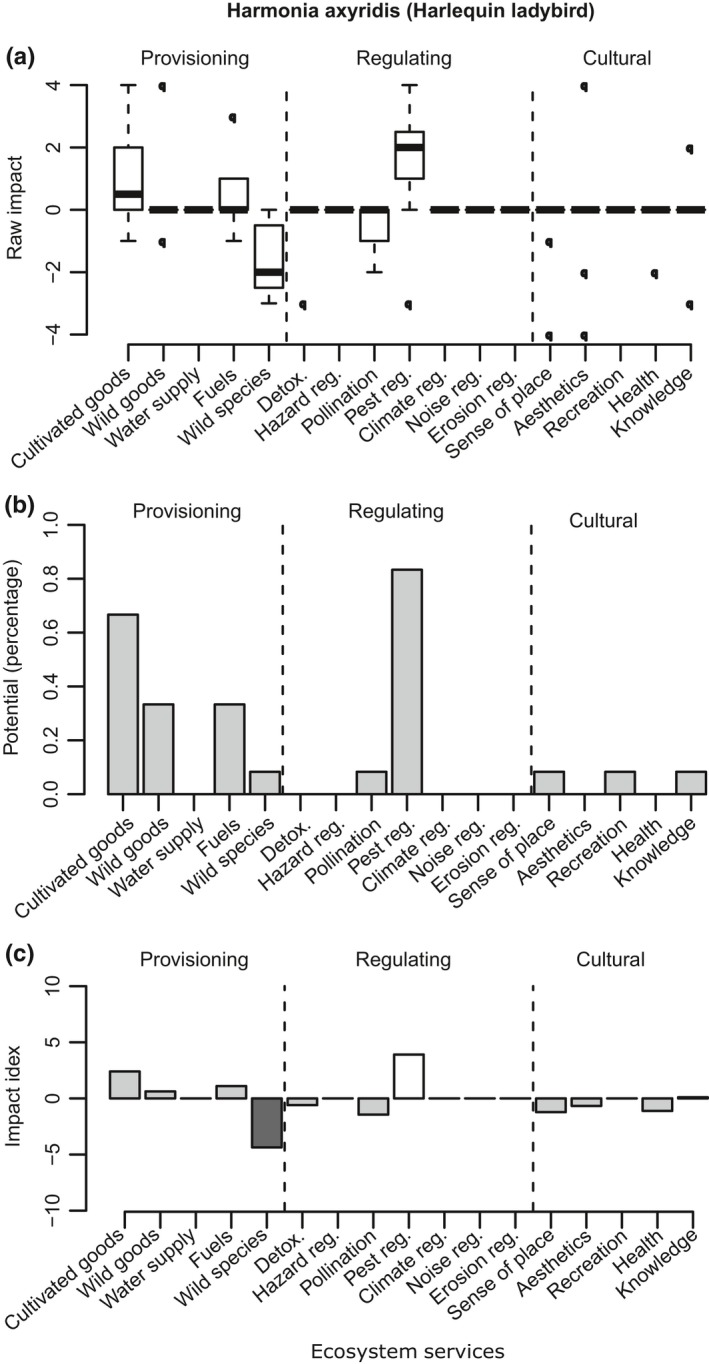
*Harmonia axyridis* (Harlequin ladybird). *N* = 12. The horizontal axis displays the ES grouped into broader categories. (a) Impact scores. Boxplot indicates the interquartile range; the band represents the median. (b) Potential. Percentage of the respondents that considered that an ES could be potentially provided by the species. (c) Impact index. White indicates strong positive impact; dark gray represents strong negative impact. Note: these results are based on the INSEAT pilot assessment form (Appendix 2)

**Figure 3:**
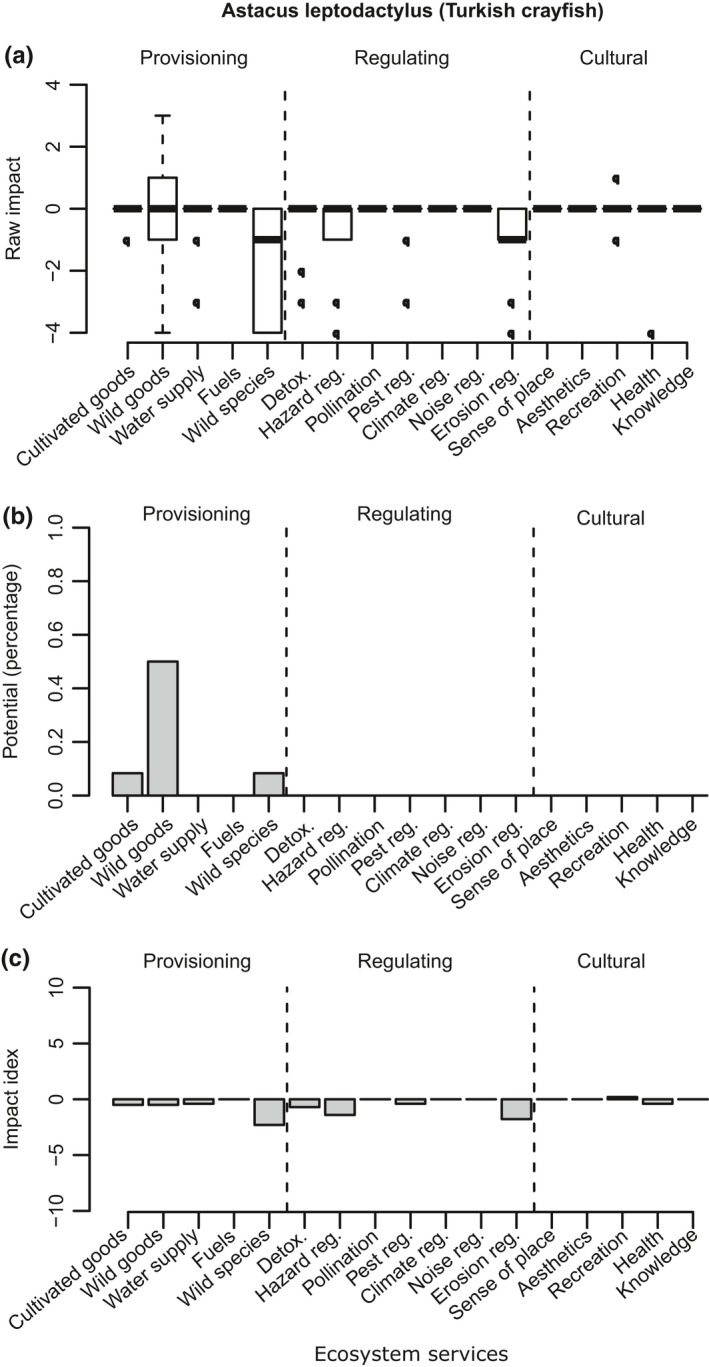
*Astacus leptodactylus* (Turkish crayfish). *N* = 12. The horizontal axis displays the ES grouped into broader categories. (a) Impact scores. Boxplot indicates the interquartile range; the band represents the median. (b) Potential. Percentage of the respondents that considered that an ES could be potentially provided by the species. (c) Impact index. Light gray indicates strong positive impact; dark gray represents strong negative impact. Note: these results are based on the INSEAT pilot assessment form (Appendix 2)

We assumed that the effect of a widely distributed species to be greater than if it were more narrowly distributed. Therefore, the “Impact Index,” was determined by weighing the *species impact* (from −4 to 4) score with its *spatial occupation* score (from 1 to 3) (i.e., Impact index = impact*occupation). The spatial occupation score of the invasive species in their non‐native range—ranging from 1 (localized occupation) to 3 (nationwide occupation)—was obtained from the respondents. Hence, *Impact index *scores range from −12 to 12: scores from −12 to −4 indicate strong negative impacts, scores from −4 to 4 indicate mild or null effects and scores from 4 to 12 indicate strong positive effects. The color code on the “Index graphs” (Figures 1b, 2b and 3b) is based on this division: dark gray denotes strong negative effect; light gray denotes mild effect; and white denotes strong positive effect.

Finally, we wanted to know the similarities and contrasts in the effects among species. This might be useful to answer ecological questions—such as “Do IAS from same taxonomic groups have similar effects, and do those effects differ between taxonomic groups?”—that may ultimately help to design management plans. Then, we used *k*‐means clustering algorithm (Hartigan & Wong, 1979) to determine the naturally occurring groups within the dataset, and the Silhouette Plot method (Appendix 3) to measure the fitness of the clustering (Kaufman & Rousseeuw, 1990).

### 2.3 Assessing species potential to provide ES

We assumed that IAS has a potential to provide ecological or cultural benefits under appropriate management (defined as any management scenario that would lead to the improvement of a particular ecosystem service provided by a species). To assess this, the respondents were asked to select a list of ES that could potentially be enhanced by the species in question under adequate management.

### 2.4 Assessing species manageability

Prioritization of cost‐effective IAS management is often essential for site managers, due to limited resources. Risk management is a tool for prioritization of IAS, used together with risk assessment. A risk management scheme, developed by Booy et al. (2017), uses seven key criteria: Effectiveness, Practicality, Cost, Impact, Acceptability, Opportunity window and Likelihood of re‐invasion.

As part of the quick IAS assessment proposed here, we developed a basic manageability assessment for assessing the feasibility of eradicating an IAS. This complements the results of the IAS effects assessment by providing a more comprehensive information about the ecology of the species in question. We based the manageability of the species on their *spreading capacity *(i.e., invasiveness), and the *management effort* (i.e., practicality—e.g., physical access and resources such as overall costs, dependent on machinery, staff and materials such as pesticides) that would be required for its eradication locally (see Booy et al., 2017).

Two semiquantitative Likert scale questions were included in the survey to obtain scores for the spreading capacity and the required management effort, respectively (Figure 1, questions 2 and 3). The scores are then presented in a scatter plot to represent the manageability of the species (Figure 6). Species on the top left corner require more resources to be eradicated than species on the bottom right corner.

### 2.5 Piloting INSEAT: Case studies

Approximately 3,864 alien species are currently established in Great Britain (Zieritz, Armas, & Aldridge, 2014). However, only 15.3% of them are considered to have negative effects on the environment or human well‐being (Roy et al., 2012). For piloting INSEAT, we selected 18 most‐studied IAS from six taxonomic groups—namely, terrestrial higher plants, mammals, aquatic crustaceans, birds, insects, and marine plants—to allow comparisons within and between groups (Appendix 4).

The respondents selected for piloting INSEAT were all IAS experts in Great Britain. We identified these respondents from the Delivering Alien Invasive Species Inventories for Europe database, as well as the relevant scientific publications. We contacted a total of 452 experts via email, requesting them to complete an anonymous online survey (surveysoftware, https://www.isurvey.soton.ac.uk/) on a voluntary basis. This pilot exercise was approved by an Ethics Committee at the University of Southampton.

All the graphical outputs and statistical analysis were performed using RStudio 3.3.1 (R Core Team, 2016), R packages “ggplot2” (Wickham, 2009), “ggrepel” (Slowikowski, 2016) and “Flexible Procedures for Clustering” (Hennig, 2015). The pilot assessment form can be found in Appendix 2; this assessment form improved after the pilot thanks to the feedback provided by the respondents and reviewers. The final assessment form is shown in Figure 1.

### 2.6 Categorizing level of confidence

We acknowledge the feedback from the testing of INSEAT that the pilot proforma lacks the capacity for the experts to validate their responses. The fact that respondents did not need to justify their answers or indicate their degree of uncertainty may strongly reduce the reliability of the assessment. Although the strength of INSEAT lies on its ability to rapidly obtain responses from a large number of experts, scores derived from this tool will inevitably have varying degree of uncertainty associated with them. In order to keep a balance between practicality and reliability, we added a section in the revised proforma asking the respondents to report the confidence level of their assessment for each ES (as High, Medium or Low; for definitions, see Figure 1). We also added a request to the respondents for information (e.g., scientific evidence, personal observations, professional opinions) that support their scores in general. Understanding the uncertainty of the responses and its implications can help to further inform IAS management decisions.

## 3 RESULTS

Our pilot survey, covering 18 IAS, was completed by 78 IAS experts in total (i.e., response rate of 17%). The average number of species completed by a respondent was 3 (95% CI = 0.41) and the average time to complete the questionnaire (the pilot version) for one species was 8.4 min (95% CI = 1.94). Each species was assessed 12.8 times on average (95% CI = 3.84), with marked variations between taxonomic groups: higher plants received a total of 75 completed assessments; mammals 47; aquatic crustaceans 45; birds 28; insects 19; and marine plants 16. The most assessed species were *Fallopia japonica* (Japanese knotweed) with 28 completed assessments, *Impatiens glandulifera* (Himalayan balsam) with 26 and *Sciurus carolinensis* (Gray squirrel) 22. The least assessed were *Frankliniella occidentalis*, (Western flower thrips) with five completed assessments, *Codium fragile* (Green sea fingers) three and *Leptoglossus occidentalis* (Western conifer seed bug) two only (Appendix 4).

Here we highlight the survey results of four IAS, showcasing how INSEAT can rapidly identify the ES enhanced or degraded by a particular IAS. The species highlighted here were chosen for their contrasting results, which help to illustrate how INSEAT can highlight variability in agreements among experts (for the results of the rest of the species, see Data Accessibility section).

**Figure 4:**
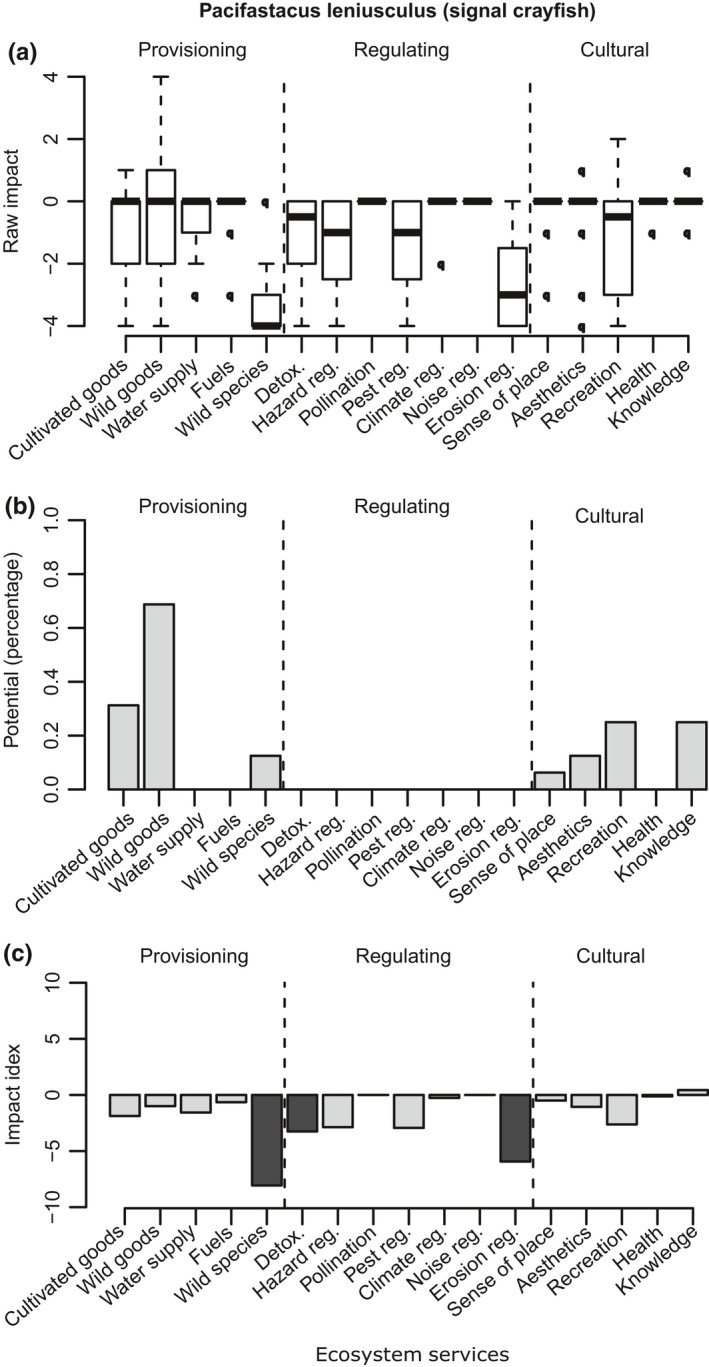
*Pacifastacus leniusculus* (Signal crayfish). *N* = 16. The horizontal axis displays the ES grouped into broader categories. (a) Impact scores. Boxplot indicates the interquartile range; the band represents the median. (b) Potential. Percentage of the respondents that considered that an ES could be potentially provided by the species. (c) Impact index. Light gray indicates strong positive impact; dark gray represents strong negative impact. Note: these results are based on the INSEAT pilot assessment form (Appendix 2)

**Figure 5:**
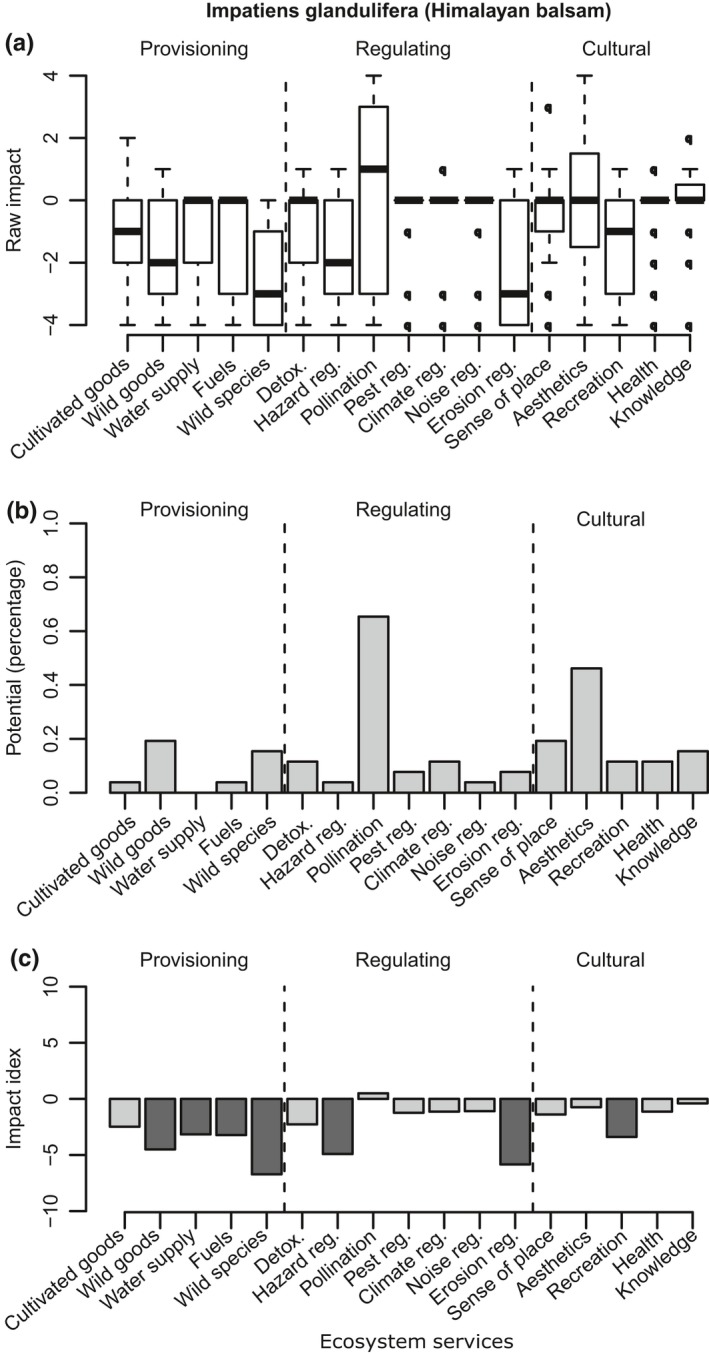
*Impatiens glandulifera* (Himalayan balsam). *N* = 26. The horizontal axis displays the ES grouped into broader categories. (a) Impact scores. Boxplot indicates the interquartile range and the band represents the median. (b) Potential. Percentage of the respondents that considered that an ES could be potentially provided by the species. (c) Impact index. Light gray indicates strong positive impact; dark gray represents strong negative impact. Note: these results are based on the INSEAT pilot assessment form (Appendix 2)

- *Harmonia axyridis *(Figure 2)—Harlequin ladybird is an Asian beetle, introduced in Europe for pest control that has accidentally arrived in Great Britain crossing the Channel together with imported vegetables. It was first recorded in Essex in 2004. Currently, it is well established in England and Wales while rapidly spreading to Scotland (Roy, 2015). This invasive species was assessed by 12 experts in this study. The experts agreed that *Harmonia axyridis *has a positive impact through its effect on pest regulation. This also has a synergistic association with other benefits such as the production of cultivated goods (Figure 2a). Furthermore, 30% of the experts considered that this ladybird is potentially beneficial for provision of fuels (i.e., beneficial for standing vegetation) and harvested wild goods (Figure 2b). However, the experts had also identified some negative effects associated with this IAS; primarily this species could adversely affect wild species diversity, or genetic diversity (with a median score of −2). Therefore, this case study demonstrates how the tool could be employed to detect important trade‐offs between the provision and loss of services associated with an invasive species (Figure 2c).
- *Astacus leptodactylus* (Figure 3)—Turkish crayfish occupies lakes, ponds, and rivers, but it has also been recorded in brackish water (Aldridge, 2016). This species was first recorded in 1975. Currently, it is well established in England with isolated populations in Wales as well. This invasive species was assessed by 12 experts. The overall effect of this species in the country is considered as “mild” as none of the effect index is higher than 3 or lower than −3 (Figure 3c). This case study, however, highlighted a discrepancy among the experts in terms of their views on the usefulness of this species used as a food source (Figure 3a). Nevertheless, 50% of the respondents indicated that there is a potential of this species to be used as a harvested wild good (Figure 3b).
- *Pacifastacus leniusculus* (Figure 4)—Interestingly, the experts’ opinions on Signal crayfish were greatly different from those of the Turkish crayfish. Hence, this case study serves as an example of how similar species are considered to have vastly different effects by the assessed experts. Assessed by 16 experts, this invasive species had negative impact index scores on wild species diversity (median score = −4), erosion regulation (median score = −3), detoxification (median score = −0.5), hazard regulation (median score = −1), pest regulation (median score = −1), and recreation (median score = −0.5) (Figure 4a). Despite the majority of the effects being negative, 70% of the experts indicated that this crayfish could potentially be used as a harvested wild good (Figure 4c).
- *Impatiens glandulifera *(Figure 5)—Himalayan balsam is an annual weed native from the Indian subcontinent. Recorded for the first time in 1851 in Great Britain, it is currently distributed through most lowland (Day, 2015). We had 26 experts assessing this species. The majority of the effects of this invasive species were considered negative (Figure 5a). The level of congruence for two particular ES is low (i.e., high uncertainty): erosion regulation (median score of −3; quartiles ranging from 0 to −4), and pollination, (median score of 1; quartiles ranging from 3 to −3). Nevertheless, the impact index scores clearly indicated that this species as highly damaging to the environment (Figure 5c).

### 3.1 Manageability and clustering analysis

**Figure 6:**
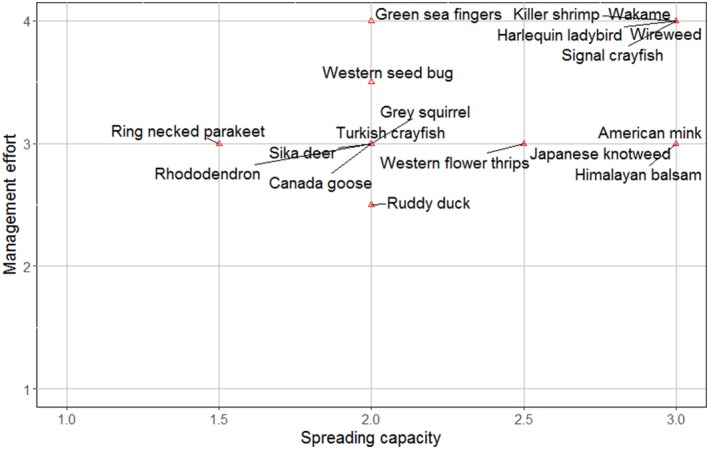
Scatter plot representing the manageability of the species. *x*‐axis represents the median of the spreading capacity; *y*‐axis represents the median of the management effort. Species in the top, right corner are the species with the lowest manageability. Note: these results are based on the INSEAT pilot assessment form (Appendix 2); the final version includes an improved definition of the management effort (Figure 1)

Overall, the manageability of all 18 IAS in this study is low, with a *management effort* median score of 3.0 (Median Absolute Deviation = 0), and *spreading capacity* median score of 2.3 (MAD = 0.74). This means that all species in this study would require a high amount of resources for their control. The species with the lowest manageability were (Figure 6) as follows: *Dikerogammarus villosus* (Killer shrimp), *Undaria pinnatifida* (Wakame), *Harmonia axyridis*, *Sargassum muticum* (Wireweed), and *Pacifastacus leniusculus*.

The clustering analysis indicated that the best number of clusters for our species sample is three, with an average silhouette width of 0.27. This silhouette width is substantially low, indicating a weak clustering structure (Appendix 3). Hence, no statistically significant cluster was found among the 18 IAS in the study (Kaufman & Rousseeuw, 1990).

## 4 DISCUSSION

Preventing IAS spread is the most cost‐effective strategy to build IAS‐free ecosystems (Richardon & Ricciardi, 2013). However, such management approach is unlikely to be 100% effective (Chornesky et al., 2005); and the ongoing rapid rates of species invasion suggest that eradication of IAS may not be economically feasible in the future. In such scenario, goals of coexistence would be more viable and realistic (Hobbs et al., 2006; Hobbs, Higgs, & Harris, 2009; Walther et al., 2009).

By using INSEAT, conservation practitioners and site managers can improve their understanding of the invasive species and their associated ecosystem service gains and losses. Such knowledge based on experts’ opinions can potentially aid in the prioritization of IAS management and the consideration of alternative management measures in decision‐making. Nevertheless, INSEAT should still be considered as a practical tool for preliminary assessments; the results of INSEAT are based on opinions of single individuals, hence they provide an initial screening of possibilities that should be further evaluated in later stages of decision‐making processes. However, the use of INSEAT could potentially pave the way for the more detailed evaluation in the future.

INvasive Species Effects Assessment Tool can highlight the level of confidence in our current knowledge of IAS, thus enabling us to pinpoint any research gaps and/or conjectures, as negative connotations of some alien species may be based on incomplete information (Bonanno, 2016; Davis, 2009; McMahon et al., 2006; Schlaepfer et al., 2011, 2012). The lack of congruence in the responses from our pilot scheme could be due to the unclear definitions of the impact scales (which we have improved after piloting). Another possible explanation for the low level of congruence in the responses could be the interpretative flexibility of the experts. It is known that opinions among experts about the valuation of IAS effects often diverged (Humair, Edwards, Siegrist, & Kueffer, 2014). This is because the notion of IAS as concepts have similar but not identical meaning to different group of experts and stakeholders; this interpretative flexibility bears the risk of introducing misunderstandings. Humair et al. (2014) urged IAS experts to acknowledge uncertainties, to engage transparently in deliberation about conflicting issues and to take the role of impartial mediators of policy alternatives rather than of issue advocates. INSEAT supports this observation, with an aspiration that our results will aid in this deliberation.

In some IAS, the direction of their effects on certain ES remained equivocal. For instance, the impact score of Himalayan balsam on pollination ranged from 3 to −3. Furthermore, the socio‐cultural attitudes of the respondents toward a particular species could also vary. This was prominently reflected by the significant variations (ranging from positive to negative) in the impact scores for cultural ES—such as “aesthetics”—in many cases. As the assessments on cultural services are dependent on personal views, it could therefore inevitably be opened to more ambiguous outcomes.

Having incorporated the positive effects provided by IAS, INSEAT provides a more comprehensive assessment of the IAS consequences across different types of ES, as opposed to focusing on the negative aspects exclusively. This will provide users new insights into the species, allowing diversification of management actions. Once the prevention measures have failed, goals of coexistence are more feasible than eradication in terms of economic resources, time and management effort (Davis, 2009; Wittenberg & Cock, 2005). Hence, these management strategies should be preferred whenever it is possible. Successful management strategies often acknowledge “that the primary and inevitable constant of the natural world is change” (Davis, 2009). Therefore, we suggest an adaptive management approach to deal with IAS (Murray & Marmorek, 2003) in which INSEAT would allow users to (a) synthesize the experts’ opinions of IAS effects; (b) collect the information that supports such opinions; and (c) explore management actions alternative to control and eradication. A re‐evaluation of known effects in the context of ES can help to bridge the link between IAS and human well‐being (Millennium Ecosystem Assessment, 2005). There are accounts of how the removal of an alien species could compromise the provision of cultural ES in a local context and lead to strong public opposition (Bennett, 2016; Bonanno, 2016; Dickie et al., 2014). Information gathered about the effects of an invasive species can be used, in combination with local knowledge, to work with stakeholders to identify the most appropriate management plan. For example, *Sciurus carolinensis* (gray squirrel)—one of the pilot species in this study—had received positive impact scores on multiple cultural ES and comments such as “for some people in the most urbanized areas, gray squirrels are their only experience of wildlife.” The removal of gray squirrel had led to strong public opposition in the past (Bremner & Park, 2007); INSEAT would have allowed wildlife managers to circumvent public outrage by identifying alternative, socially acceptable squirrel management plans.

One useful feature of INSEAT is that it could highlight the potential benefits that an invasive species could provide under appropriate management (Figures 1b, 2b, 3b and 4b). Under certain climate change scenarios, some non‐native species have even been considered necessary to assure local ecosystem function continuity (Lin & Petersen, 2013; Walther et al., 2009). Cases of IAS providing refuge for native species have also been reported (Chiba, 2010). We therefore argue that consideration of management alternatives to the *status quo* can help to mitigate negative impacts while taking advantage of the alien species; IAS can be a valuable resource in their own right, and management actions that take advantage of their potential benefits could be fruitful. A comment from an expert on Cervus nippon (Sika deer) supported our case: “if deer numbers could be controlled, perhaps by bringing back the Lynx, there are definitely positive benefits.” Another example that justified the usefulness of IAS is that both Turkish and Signal crayfish scored high in their potential as wild food resource, with 50% and 70% of experts in agreement, respectively. Management measures that include harvesting of wild populations could decrease their numbers, diminish their negative effect on other services and increase the cultural values that are associated with the harvest. However, when such management is considered, it should be done with precaution: many examples in the literature illustrate the risk of exploiting invasive species, for example, promoting the intentional introduction of fish and crayfish into areas where the species was not present (McLaughlan & Aldridge, 2013; McLaughlan et al., 2014). In such cases, site managers could explore if recreational harvest accompanied by IAS awareness and education is a possible solution for preventing unintended consequences of exploiting invasive species.

Invasive alien species management involves an estimation of the resources required for effective control. INSEAT allows users to visualize the level of manageability of an invasive species (Figure 6), thus providing a preliminary assessment of feasibility of IAS management. To enhance the efficacy of a control measure, the tool also allows users to distinguish groups of IAS with similar effects. Our clusters were not statistically significant for all pilot species; this is not surprising given that they were from six different taxonomic groups. To be useful, the clustering analysis should include invasive species from the same taxon (e.g., avian) only.

Finally, we believe that INSEAT can be applied on a user‐friendly web interface and adapted as an online survey which can be completed rapidly. It can be adapted to different geographical or political regions; and the results are visually informative and self‐explanatory for site managers and stakeholders.

### 4.1 Limitations and future perspectives

INSEAT—as a rapid assessment tool—inevitably has limitations. It does not deal with complex ecological interactions, scale‐dependent effects, intricate ecological context, and spread mechanisms. INSEAT could not provide answers to the many complexities in ES science. For example, it is beyond the scope of the tool to address sustainability and resilience of the ES associated with IAS. Nevertheless, INSEAT should complement other risk assessments (e.g., Booy et al., 2017) and be used to build awareness, detect knowledge gaps and aid in the design of alternative management strategies. In fact, a bridge between INSEAT and EICAT, which evaluates, compares, and predicts the magnitudes of the environmental impacts of different IAS taxa (Hawkins et al., 2015), would be beneficial for both IAS management and policy. Decision‐makers could then evaluate all the knowledge available, while exploring management alternatives, by focusing on the functional role rather than on the origin of the species (Bonanno, 2016).

## ACKNOWLEDGMENTS

This work took place under the “Attaining Sustainable Services from Ecosystems using Trade‐off Scenarios” project (ASSETS, http://espa-assets.org/; NE‐J002267‐1), funded with support from the United Kingdom's Ecosystem Services for Poverty Alleviation program (ESPA, http://www.espa.ac.uk). ESPA receives its funding from the Department for International Development (DFID), the Economic and Social Research Council (ESRC) and the Natural Environment Research Council (NERC).

## CONFLICT OF INTEREST

None declared.

## AUTHORS’ CONTRIBUTIONS

R.M.‐C. and K.S.‐H.P. conceived the ideas; R.M.‐C. and K.S.‐H.P. designed the tool with the input from E.J., S.W. and P.V.; R.M.‐C. collected the data; R.M.‐C. and A.P.‐D. analyzed the data; and R.M.‐C. wrote the manuscript, with all co‐authors contributing to the earlier drafts.

## Figures and Tables

**Figure A3:**
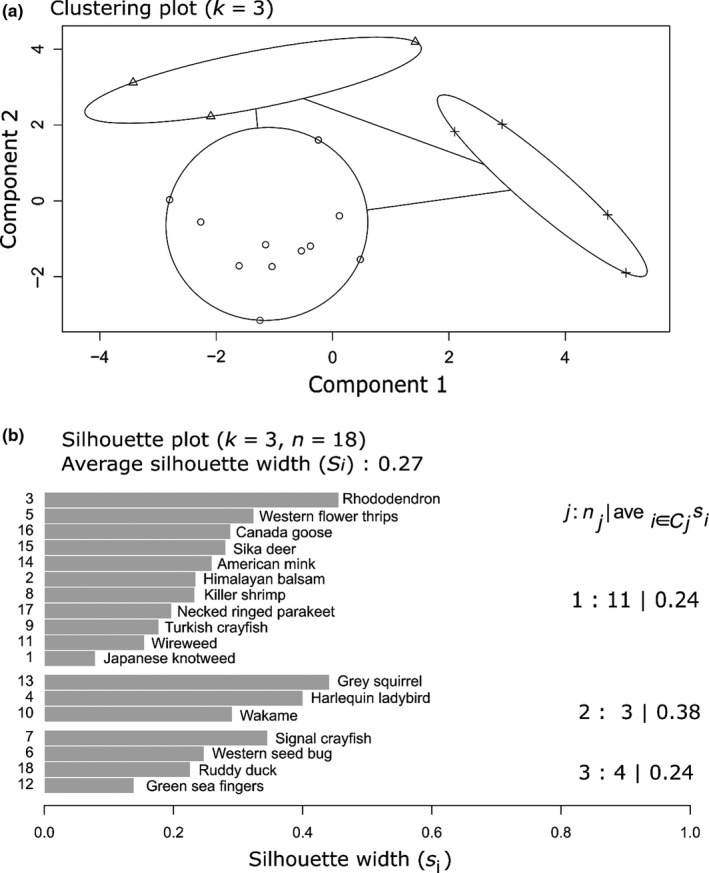
*K*‐mean clustering plot and Silhouette plot obtained for the species of the case studies. (a) Clustering plot. The 16 case studies are represented in two dimensions and grouped into three clusters. Components 1 and 2 explain 55.03% of the point variability. (b) Silhouette plot used to select the best number of clusters. From all the possible number of clusters, *k* = 3 has the highest Average Silhouette Width (*S*_(_ *_i_*_)_ = 0.27). This low value indicates weak cohesion among the data points. *j*: cluster; *n_j_*: number objects (species) in each cluster; ave*_i_*_∈C_ *_j_* *s_i_*: average width of each cluster

## Data Availability

Data generated by INSEAT for the case studies will be available through DRYAD, and include the following: (1) the raw data, consisting on the online surveys as downloaded from https://www.isurvey.soton.ac.uk, (2) the data file used to analyze the results, obtained by cleaning the raw data file and (3) R files required to build the graphical outputs designed for INSEAT. For each species, the graphical outputs generated (impact scores, potential and impact index graphs) will be shared through FIGSHARE.

## APPENDIX 1

Correspondence table of the integrated classification and ecosystem services proposed for this study. We integrated three commonly accepted classification schemes from the Millennium Ecosystem Assessment (Millennium Ecosystem Assessment, 2005), the UK National Ecosystem Assessment (UK NEA; Mace et al., 2011), and The Economics of Ecosystem and Biodiversity (TEEB, 2016). The colors indicate the categories of services: provisioning (green), regulating (orange) and cultural (red). N/A indicates that the category was “not available” in a particular classification scheme.

**Table:**

	This study	UK NEA	MA	TEEB
Provisioning	Crops or livestock	Crops, livestock, fish	Food	Food
	Harvested wild goods	N/A	Ornamental resources	N/A
		N/A	N/A	Medicinal resources
	Water supply	Water supply	Freshwater	Freshwater
	Trees, standing vegetation, peat	Trees, standing vegetation, peat	Biochemicals	Raw materials
			Fiber	
	Wild species diversity	Wild species diversity	Genetic resources	N/A
		Wild species diversity		
Regulating	Detoxification and purification in soils air and water	Detoxification and purification in soils, air, and water	Water purification and waste treatment	Local climate and air quality
			Air quality regulation	Waste‐water treatment
	Hazard regulation	Hazard regulation	Natural hazard regulation	Moderation of extreme events
	Pollination	Pollination	Pollination	Pollination
	Disease and pest regulation	Disease and pest regulation	Pest regulation	Biological control
			Disease regulation	
	Climate regulation	Climate regulation	Climate regulation	Carbon sequestration and storage
	Noise regulation	Noise regulation	N/A	N/A
	Erosion regulation	N/A	Erosion regulation	Erosion prevention and maintenance of soil fertility
Cultural	Spiritual experience and sense of place	Environmental settings	Spiritual and religious values	Spiritual experience and sense of place
			Sense of place	
	Aesthetic appreciation and inspiration for culture, art, design		Cultural heritage values	Aesthetic appreciation and inspiration for culture, art, design
			Cultural diversity	
			Aesthetic values	
			Inspiration	
	Recreation and tourism		Recreation and ecotourism	Recreation and mental and physical health
				Tourism
	Mental and physical health		Social relations	Recreation and mental and physical health
	Knowledge systems and educational values		Educational values	N/A
			Knowledge systems	

## APPENDIX 2

### INSEAT (FIRST VERSION) FOR PILOTING PHASE

Asterisks indicate where modifications have been made for the final version; the improvements were based on the feedback provided by respondents of the survey and reviewers.

**Table:**

[*Species name* – Common name]		
If you feel that you do not have the knowledge to assess this species, please proceed with the next species		
**Question 1. Spatial occupation**		
*Score*	Please describe the *current invasion stage* of this species in [Country/area]	
*1*	Local—the species is found in a single site or covers a small area of the [country/area], <10% of the area of the [country/area]	
*2*	Regional—populations are present in between 10% and 75% of the area of the [country/area]	
*3*	National—populations are present in more than 75% of the area of the [country/area]	
–	I do not know	
^(*)^		
**Question 2. Spreading capacity**		
*Score*	Please describe the *spreading capacity* of this species	
*1*	Low potential—the species spreads slowly	
*2*	Medium/moderate potential—the species spreads rapidly but does not double its range in <10 years	
*3*	High potential—the species spreads rapidly, doubling its range in <10 years	
–	I do not know	
**Question 3. Management effort** ^(^ **^**^** ^)^		
*Score*	Please select the management effort necessary to *eradicate* this species	
*1*	Unmanageable—management measures cannot control it	
*2*	High—can be controlled with intensive management	
*3*	Medium—can be eradicated with periodic management	
*4*	Low—successfully eradicated with no ongoing management	
*–*	I do not know	
**Question 4. Ecosystem services impact assessment**		
We have designed a semi‐quantitative scale that assesses both positive and negative effects of this species. Each score is defined as:		
*Score*	Impact score definition^(***)^	
*4*	**Intense** positive impact to the ecosystem service; the effect is **non‐permanent**	
*3*	**Noticeable** positive impact to the ecosystem service but **short‐lasting**	
*2*	Positive impact on the ecosystem service is **too small** to be significant	
*1*	**No impacts** detectable/ecosystem services **not applicable** to this species	
*0*	Negative impact on the ecosystem service is **too small** to be significant	
*−1*	**Noticeable** negative impact to the ecosystem service; damage is **short‐lasting**	
*−2*	**Intense **negative impact to the ecosystem service; damage is **non‐permanent**	
*−3*	Negative impact to the ecosystem service is both **intense** and **lasting**	
*−4*	**Intense** positive impact to the ecosystem service; the effect is **non‐permanent**	
*–*	I don't know	
Please score the **general** impact of this species on each ecosystem service and indicate the confidence using the guidance table in Question 1:		
		Score
**Provisioning services. *These are the products that people obtain from ecosystems*.**		
Crops or livestock *(i.e., provision of food)*		
Harvested wild goods *(i.e., ornamental/medicinal resources/wild game)*		
Trees, standing vegetation, peat *(i.e., fuels and construction material)*		
Water supply *(i.e., supply of local water)*		
Wild species diversity *(genetic diversity for animal and plant breeding)*		
**Regulating services*. These are the benefits obtained from regulation of ecosystem processes*.**		
Detoxification and purification in soils, air and water		
Climate regulation *(i.e., local (e.g., temperature and precipitation) or global (e.g., carbon sequestration) regulation)*		
Hazard regulation *(i.e., moderation of extreme events as floods or storms)*		
Pollination		
Noise regulation		
Erosion regulation *(i.e., Erosion prevention and maintenance of soil fertility)*		
**Cultural services.** ***These are the nonmaterial benefits obtained from ecosystems***		
Spiritual experience and sense of place *(i.e., religious meaning or sense of belonging)*		
Aesthetic appreciation and inspiration for culture, art, design		
Recreation and tourism		
Mental and physical health		
Knowledge systems and educational values *(i.e., types of knowledge and basis for education)*		
**Question 5. One additional question**		
Let's suppose that the negative impacts of this species on the environment can be mitigated with an appropriate management of its wild populations		
Do you think this species would then have the **potential** to provide any of the following benefits? Please, choose *yes* for the ecosystem services that can be improved		
[List of ecosystem services]		
**Comments^(****)^**		
Please give any comments regarding this assessment or about the potential benefits that this species can provide (e.g., management needed or limitations)		
[Comment box]		

^(*) ^To determine uncertainty, the revised proforma asks respondents to report the level of confidence in their assessments, and to provide information that support their scores.

^(**) ^Management effort. The scale used to define the management effort was improved, inspired by the work of Booy et al. (2017).

^(***) ^In the revised version, the impact categories are better defined, and use terminologies that avoid making judgements on the effect of IAS on the ES. The new version also includes the option “Data deficient” for acknowledging that the lack of knowledge is because of no existing data or evidence, rather than the unawareness of an individual.

^(****) ^Question 6 was added to gather information that support the expert's responses.

## APPENDIX 3

### *K*‐MEANS CLUSTERING

Best number of clusters and interpretation of the results.

Clustering algorithms find naturally occurring groups in a dataset. The *K*‐means method divides the data points into “*k*” number of groups in which the sum of squares from these points to the clusters center is minimized (Hartigan & Wong, 1979). To select the best “*k*” (number of clusters), the Silhouette Plot method is widely used. A Silhouette Plot is a representation of the cohesion among the points of a cluster and the separation between the points of different clusters (Figure A3b) (Rousseeuw, 1987). Using the notation in the Figure, the *silhouette width* (*s_i_*) is a representation of the suitability of the object to belong to a cluster (*j*), and the *silhouette of a cluster* is a plot of the of the *s_i_* of all the objects (*n_j_*) of the cluster ranked in decreasing order. The *average silhouette plot width* (*S_i_*) is the average of the *s_i_* of all the clusters and can be used to measure of fitness of the clustering. Values <0.25 indicate that no substantial structure has been found; 0.26–0.50 indicate that the structure is weak and could be artificial; 0.51–0.70 indicate that a reasonable structure has been found, and from 0.71 to 1.0 indicate a strong structure. From all the average silhouette widths of all “*k*,” the optimum (closest to 1) indicate the “best” number of “*k*,” that is, the most “naturally” occurring number of clusters.

When interpreting the graph, one should attend both to the *average silhouette plot width* (*S_i_*) to find the “best” *k* and the *silhouette width* (*s_i_*) of each cluster for the validation of consistency: to identify outliers and objects that lie well within their cluster of the ones are merely somewhere in between clusters. For extended information check “Chapter 2. Partitioning Around Medoids (Program PAM)” from “Finding Groups in Data: An Introduction to Cluster Analysis” (Kaufman & Rousseeuw, 1990).

## APPENDIX 4

### SPECIES SELECTION PROCEDURE.

From the 282 IAS, we selected those that were chosen for factsheets by the GB‐Non‐Native Species Information Portal (GB‐NNSIP). These factsheets contain additional information of well‐known, highly damaging species. This way, we ensured that species contained in this list were well‐studied. The list was reduced to 99 species.

To further reduce the IAS number a bibliographic search was performed in Web of Science. We recorded the number of entries per species with the following search‐string: |<"Species"> OR <"Common name">| *AND* |UK OR "United Kingdom" OR GB OR "Great Britain"| *AND* |"non‐native" OR alien OR invasive|. Three species of six taxonomical groups with most papers were selected for this study. The final list of IAS included in this study includes 18 species.

**Table A4:** Final list of species included in the online assessment. No. entries in Web of Science refers to the number of entries in Web of Science on August 2016; No. times assessed refers to the number of times the species was evaluated by the respondents of the survey

Species name	Common name	No. entries in Web of Science	No. times assessed
Higher plants			
*Fallopia japonica*	Japanese knotweed	22	28
*Impatiens glandulifera*	Himalayan balsam	18	26
*Rhododendron ponticum*	Rhododendron	8	21
Insects			
*Harmonia axyridis*	Harlequin ladybird	11	12
*Frankliniella occidentalis*	Western flower thrips	2	5
*Leptoglossus occidentalis*	Western conifer seeds bug	1	2
Aquatic crustaceans			
*Pacifastacus leniusculus*	Signal crayfish	20	16
*Dikerogammarus villosus*	Killer shrimp	15	17
*Astacus leptodactylus*	Turkish crayfish	4	12
Marine plants			
*Undaria pinnatifida*	Japanese kelp/Wakame	4	28
*Sargassum muticum*	Japanese wireweed	3	6
*Codium fragile*	Green sea fingers	1	3
Vertebrates‐mammal			
*Sciurus carolinensis*	Gray squirrel	31	22
*Mustela vison*	American mink	24	15
*Cervus nippon*	Sika deer	4	10
Vertebrates‐avian			
*Psittacula krameri*	Neck‐ringed parakeet	6	10
*Branta canadensis*	Canada goose	3	8
*Oxyura jamaicensis*	Ruddy duck	3	10

## References

[ece35020-bib-0001] Aldridge, D. (2016). Non‐native invasive species factsheets: Turkish Crayfish (*Astacus leptodactylus*). GB Non‐Native Species Secretariat, United Kingdom Retrieved from http://www.nonnativespecies.org/factsheet/factsheet.cfm?speciesId=381.

[ece35020-bib-0002] Bacher, S. , Blackburn, T. M. , Essl, F. , Genovesi, P. , Heikkilä, J. , Jeschke, J. M. , … Martinou, A. F. (2018). Socio‐economic impact classification of alien taxa (SEICAT). Methods in Ecology and Evolution, 9, 159–168.

[ece35020-bib-0003] Bennett, E. M. , Peterson, G. D. , & Gordon, L. J. (2009). Understanding relationships among multiple ecosystem services. EcologyLetters, 12, 1394–1404. 10.1111/j.1461-0248.2009.01387.x 19845725

[ece35020-bib-0004] Bennett, N. J. (2016). Using perceptions as evidence to improve conservation and environmental management. Conservation Biology, 30(3), 582–592. 26801337 10.1111/cobi.12681

[ece35020-bib-0005] Bonanno, G. (2016). Alien species: To remove or not to remove? That is the question. Environmental Science & Policy, 59, 67–73.

[ece35020-bib-0006] Boonman‐Berson, S. , Turnhout, E. , & van Tatenhove, J. (2014). Invasive species: The categorization of wildlife in science, policy, and wildlife management. Land Use Policy, 38, 204–212.

[ece35020-bib-0007] Booy, O. , Mill, A. C. , Roy, H. E. , Hiley, A. , Moore, N. , Robertson, P. , … Campbell, S. (2017). Risk management to prioritise the eradication of new and emerging invasive non‐native species. Biological Invasions, 19(8), 2401–2417.

[ece35020-bib-0008] Bremner, A. , & Park, K. (2007). Public attitudes to the management of invasive non‐native species in Scotland. Biological Conservation, 139, 306–314.

[ece35020-bib-0009] Byers, J. E. (2002). Impact of non‐indigenous species on natives enhanced by anthropogenic alteration of selection regimes. Oikos, 97, 449–458.

[ece35020-bib-0010] Chiba, S. (2010). Invasive non‐native species’ provision of refugia for endangered native species. Conservation Biology, 24, 1141–1147. 20184648 10.1111/j.1523-1739.2010.01457.x

[ece35020-bib-0011] Chornesky, E. A. , Bartuska, A. M. , Aplet, G. H. , Britton, K. O. , Cummings‐Carlson, J. , Davis, F. W. , … Hansen, A. J. (2005). Science priorities for reducing the threat of invasive species to sustainable forestry. BioScience, 55, 335–348.

[ece35020-bib-0012] Çinar, M. E. , Arianoutsou, M. , Zenetos, A. , & Golani, D. (2014). Impacts of invasive alien marine species on ecosystem services and biodiversity: A pan‐European review. Aquatic Invasions, 9, 391–423.

[ece35020-bib-0013] Convention on Biological Diversity (2008). Alien Species that Threaten Ecosystems, Habitats or Species. Article 8(h), Convention on Biological Diversity, United Nations.

[ece35020-bib-0014] Wittenberg, R. , & Cock, M. J. (2001). Invasive alien species: A toolkit of best prevention and management practices. Wallingford, UK: CAB International.

[ece35020-bib-0015] Cook, D. , & Proctor, W. (2007). Assessing the threat of exotic plant pests. Ecological Economics, 63, 594–604.

[ece35020-bib-0016] Cooke, R. M. (1991). Experts in uncertainty: Opinion and subjective probability in science. New York, NY: Oxford University Press.

[ece35020-bib-0017] Corbin, J. D. , & D'Antonio, C. M. (2004). Competition between native perennial and exotic annual grasses: Implications for an historical invasion. Ecology, 85, 1273–1283. 10.1890/02-0744

[ece35020-bib-0018] Costanza, R. , de Groot, R. , Braat, L. , Kubiszewski, I. , Fioramonti, L. , Sutton, P. , … Grasso, M. (2017). Twenty years of ecosystem services: How far have we come and how far do we still need to go? Ecosystem Services, 28, 3918–16.

[ece35020-bib-0019] Daily, G. C. (1997). Nature's services: Societal dependence on natural ecosystems. Washington, DC: Island Press.

[ece35020-bib-0020] Davis, M. A. (2009). Invasion biology. Oxford, UK: Oxford University Press.

[ece35020-bib-0021] Day, P. (2015). Non‐native invasive species factsheets: Himalayan Balsam (Impatiens glandulifera). United Kingdom: GB Non‐Native Species Secretariat Retrieved from http://www.nonnativespecies.org/factsheet/factsheet.cfm?speciesId=1810.

[ece35020-bib-0022] Dickie, I. A. , Bennett, B. M. , Burrows, L. E. , Nunez, M. A. , Peltzer, D. A. , Porté, A. , … Van Wilgen, B. W. (2014). Conflicting values: Ecosystem services and invasive tree management. Biological Invasions, 16, 705–719.

[ece35020-bib-0023] Didham, R. K. , Tylianakis, J. M. , Hutchison, M. A. , Ewers, R. M. , & Gemmell, N. J. (2005). Are invasive species the drivers of ecological change? Trends in Ecology & Evolution, 20, 470–474. 16701420 10.1016/j.tree.2005.07.006

[ece35020-bib-0024] Eviner, V. T. , Garbach, K. , Baty, J. H. , & Hoskinson, S. A. (2012). Measuring the effects of invasive plants on ecosystem services: Challenges and prospects. Invasive Plant Science and Management, 5, 125–136.

[ece35020-bib-0025] Ewel, J. J. , & Putz, F. E. (2004). A place for alien species in ecosystem restoration. Frontiers in Ecology and the Environment, 2, 354–360.

[ece35020-bib-0026] Genovesi, P. , & Monaco, A. (2013). Guidelines for addressing invasive species in protected areas In Foxcroft L. C., Pyšek P., Richardson D. M. & Genovesi P. (Eds.) Plant invasions in protected areas (pp. 487–506). Dordrecht, the Netherlands: Springer.

[ece35020-bib-0027] Gurevitch, J. , & Padilla, D. K. (2004). Are invasive species a major cause of extinctions? Trends in Ecology & Evolution, 19, 470–474. 16701309 10.1016/j.tree.2004.07.005

[ece35020-bib-0028] Haines‐Young, R. , & Potschin, M. (2012). Common international classification of ecosystem services (CICES, Version 4.1). European Environment Agency, 33.

[ece35020-bib-0029] Hartigan, J. A. , & Wong, M. A. (1979). Algorithm AS 136: A k‐means clustering algorithm. Journal of the Royal Statistical Society. Series C (Applied Statistics), 28, 100–108.

[ece35020-bib-0030] Hawkins, C. L. , Bacher, S. , Essl, F. , Hulme, P. E. , Jeschke, J. M. , Kühn, I. , … Rabitsch, W. (2015). Framework and guidelines for implementing the proposed IUCN Environmental Impact Classification for Alien Taxa (EICAT). Diversity and Distributions, 21, 1360–1363.

[ece35020-bib-0031] Hemming, V. , Burgman, M. A. , Hanea, A. M. , McBride, M. F. , & Wintle, B. C. (2018). A practical guide to structured expert elicitation using the IDEA protocol. Methods in Ecology and Evolution, 9(1), 169–180.

[ece35020-bib-0032] Hennig, C. (2015). fpc: Flexible procedures for clustering. R package version 2.1-10. Retrieved from https://CRAN.R-project.org/package=fpc

[ece35020-bib-0033] Hobbs, R. J. , Arico, S. , Aronson, J. , Baron, J. S. , Bridgewater, P. , Epstein, C. V. A. , … D. (2006). Novel ecosystems: Theoretical and management aspects of the new ecological world order. Global Ecology and Biogeography, 15, 3918–7.

[ece35020-bib-0034] Hobbs, R. J. , Higgs, E. , & Harris, J. A. (2009). Novel ecosystems: Implications for conservation and restoration. Trends in Ecology & Evolution, 24, 599–605. 19683830 10.1016/j.tree.2009.05.012

[ece35020-bib-0035] Hulme, P. E. , Pyšek, P. , Nentwig, W. , & Vilà, M. (2009). Will threat of biological invasions unite the European Union. Science, 324, 40–41. 19342572 10.1126/science.1171111

[ece35020-bib-0036] Humair, F. , Edwards, P. J. , Siegrist, M. , & Kueffer, C. (2014). Understanding misunderstandings in invasion science: Why experts don't agree on common concepts and risk assessments. NeoBiota, 20, 3918.

[ece35020-bib-0037] Kaufman, L. , & Rousseeuw, P. J. (1990). Finding groups in data: An introduction to cluster analysis (Vol. 344). New York, NY: John Wiley & Sons.

[ece35020-bib-0038] Kremen, C. (2005). Managing ecosystem services: What do we need to know about their ecology? EcologyLetters, 8, 468–479. 10.1111/j.1461-0248.2005.00751.x 21352450

[ece35020-bib-0039] Kull, C. A. , Shackleton, C. M. , Cunningham, P. J. , Ducatillon, C. , Dufour‐Dror, J. M. , Esler, K. J. , … Midgley, S. J. (2011). Adoption, use and perception of Australian acacias around the world. Diversity and Distributions, 17, 822–836.

[ece35020-bib-0040] Levine, J. M. , Vila, M. , Antonio, C. M. , Dukes, J. S. , Grigulis, K. , & Lavorel, S. (2003). Mechanisms underlying the impacts of exotic plant invasions. Proceedings of the Royal Society of London B: Biological Sciences, 270, 775–781. 10.1098/rspb.2003.2327 PMC1691311 12737654

[ece35020-bib-0041] Lin, B. B. , & Petersen, B. (2013). Resilience, regime shifts, and guided transition under climate change: Examining the practical difficulties of managing continually changing systems. Ecology and Society, 18, 28–37. 10.5751/ES-05128-180128

[ece35020-bib-0042] Liu, S. , Proctor, W. , & Cook, D. (2010). Using an integrated fuzzy set and deliberative multi‐criteria evaluation approach to facilitate decision‐making in invasive species management. Ecological Economics, 69, 2374–2382.

[ece35020-bib-0043] MacDougall, A. S. , & Turkington, R. (2005). Are invasive species the drivers or passengers of change in degraded ecosystems? Ecology, 86, 42–55.

[ece35020-bib-0044] Mace, G. M. , Bateman, I. , Albon, S. , Balmford, A. , Brown, C. , Church, A. , … Winn , J. (2011). UK National Ecosystem assessment (UK NEA). Technical report. Chapter 2. Conceptual framework and methodology. United Kingdom.

[ece35020-bib-0045] McLaughlan, C. , & Aldridge, D. C. (2013). Cultivation of zebra mussels (*Dreissena polymorpha*) within their invaded range to improve water quality in reservoirs. Water Research, 47(13), 4357–4369. 10.1016/j.watres.2013.04.043 23764587

[ece35020-bib-0046] McLaughlan, C. , Gallardo, B. , & Aldridge, D. (2014). How complete is our knowledge of the ecosystem services impacts of Europe's top 10 invasive species? Acta Oecologica, 54, 119–130.

[ece35020-bib-0047] McMahon, S. M. , Fukami, T. , & Cadotte, M. W. (2006). Conceptual ecology and invasion biology: Reciprocal approaches to nature. Dordrecht, NL: Springer.

[ece35020-bib-0048] Meyerson, L. A. , & Mooney, H. A. (2007). Invasive alien species in an era of globalization. Frontiers in Ecology and the Environment, 5, 199–208.

[ece35020-bib-0049] Millennium Ecosystem Assessment (2005). Ecosystems and human well‐being. Washington, DC: Millennium Ecosystem Assessment.

[ece35020-bib-0050] Murray, C. , & Marmorek, D. (2003). Adaptive management and ecological restoration In Freiderici P. (Ed.), Ecological restoration of Southwestern ponderosa pine forests (pp. 417–428). Washington, DC: Island Press.

[ece35020-bib-0051] Naidoo, R. , Balmford, A. , Costanza, R. , Fisher, B. , Green, R. E. , Lehner, B. , … Ricketts, T. H. (2008). Global mapping of ecosystem services and conservation priorities. Proceedings of the National Academy of Sciences of the United States of America, 105, 9495–9500. 18621701 10.1073/pnas.0707823105 PMC2474481

[ece35020-bib-0052] Nentwig, W. , Bacher, S. , Pyšek, P. , Vilà, M. , & Kumschick, S. (2016). The generic impact scoring system (GISS): A standardized tool to quantify the impacts of alien species. Environmental Monitoring and Assessment, 188, 3918–13. 10.1007/s10661-016-5321-4 27129597

[ece35020-bib-0053] O'Hagan, A. , Buck, C. E. , Daneshkhah, A. , Eiser, J. R. , Garthwaite, P. H. , Jenkinson, D. J. , … Rakow, T. (2006). Uncertain judgements: Eliciting expert probabilities. Chichester, UK: Wiley.

[ece35020-bib-0054] Peh, K.‐S.‐H. , Balmford, A. , Birch, J. C. , Brown, C. , Butchart, S. H. M. , Daley, J. , … Millett, J. (2015). Potential impact of invasive alien species on ecosystem services provided by a tropical forested ecosystem: A case study from Montserrat. Biological Invasion, 17, 461–475.

[ece35020-bib-0055] Pejchar, L. , & Mooney, H. A. (2009). Invasive species, ecosystem services and human well‐being. Trends in Ecology & Evolution, 24, 497–504. 19577817 10.1016/j.tree.2009.03.016

[ece35020-bib-0056] Pyšek, P. , & Richardson, D. M. (2010). Invasive species, environmental change and management, and health. Annual Review of Environment and Resources, 35, 25–55.

[ece35020-bib-0057] R Core Team (2016). R: A language and environment for statistical computing. Vienna, Austria: R Foundation for Statistical Computing.

[ece35020-bib-0058] Richardson, D. M. , & Pyšek, P. (2008). Fifty years of invasion ecology–the legacy of Charles Elton. Diversity and Distributions, 14, 161–168. 10.1111/j.1472-4642.2007.00464.x

[ece35020-bib-0059] Richardson, D. M. , & Ricciardi, A. (2013). Misleading criticisms of invasion science: A field guide. Diversity and Distributions, 19(12), 1461–1467.

[ece35020-bib-0060] Rousseeuw, P. J. (1987). Silhouettes: A graphical aid to the interpretation and validation of cluster analysis. Journal of Computational and Applied Mathematics, 20, 53–65.

[ece35020-bib-0061] Roy, H. E. , Bacon, J. , Beckmann, B. , Harrower, C. A. , Hill, M. O. , Isaac, N. J. , … Musgrove, A. (2012). Non‐Native Species in Great Britain: establishment, detection and reporting to inform effective decision making. Defra report July.

[ece35020-bib-0062] Roy, H. E. (2015). Non‐native invasive species factsheet: Harlequin ladybird (*Harmonia axyridis*). United Kingdom: GB Non‐Native Species Secretariat Retrieved from http://www.nonnativespecies.org/factsheet/factsheet.cfm?speciesId=1668.

[ece35020-bib-0063] Roy, H. E. , Rabitsch, W. , Scalera, R. , Stewart, A. , Gallardo, B. , Genovesi, P. , … Branquart, E. (2018). Developing a framework of minimum standards for the risk assessment of alien species. Journal of Applied Ecology, 55, 526–538.

[ece35020-bib-0064] Roy, H. , Schonrogge, K. , Hannah, D. , Jodey, P. , Branquart, E. , Vanderhoeven, S. , … Stewart, A. (2014). Invasive alien species – framework for the identification of invasive alien species of EU concern. Brussels, Belgium: European Commission, 298 pp.

[ece35020-bib-0065] Schlaepfer, M. A. , Sax, D. F. , & Olden, J. D. (2011). The potential conservation value of non‐native species. Conservation Biology, 25, 428–437. 21342267 10.1111/j.1523-1739.2010.01646.x

[ece35020-bib-0066] Schlaepfer, M. A. , Sax, D. F. , & Olden, J. D. (2012). Toward a more balanced view of non‐native species. Conservation Biology, 26, 1156. 23082954 10.1111/j.1523-1739.2012.01948.x

[ece35020-bib-0067] Seabloom, E. W. , Harpole, W. S. , Reichman, O. , & Tilman, D. (2003). Invasion, competitive dominance, and resource use by exotic and native California grassland species. Proceedings of the National Academy of Sciences of the United States of America, 100, 13384–13389. 14595028 10.1073/pnas.1835728100 PMC263823

[ece35020-bib-0068] Shackleton, C. M. , McGarry, D. , Fourie, S. , Gambiza, J. , Shackleton, S. E. , & Fabricius, C. (2007). Assessing the effects of invasive alien species on rural livelihoods: Case examples and a framework from South Africa. Human Ecology, 35, 113–127. 10.1007/s10745-006-9095-0

[ece35020-bib-0069] Simberloff, D. (2000).Non-indigenous species: A global threat to biodiversity and stability In Raven P. & Williams T. (Eds.), Nature and human society: The quest for a sustainable world. (pp. 325–334). Washington, DC: National Academy Press.

[ece35020-bib-0070] Slobodkin, L. B. (2001). The good, the bad and the reified. Evolutionary Ecology Research, 3, 91–105.

[ece35020-bib-0071] Slowikowski, K. (2016). ggrepel: Repulsive text and label geoms for ggplot2. R package version 0.5. Retrieved from https://CRAN.R-project.org/package=ggrepel

[ece35020-bib-0072] Sutherland, W. J. , & Burgman, M. (2015). Policy advice: Use experts wisely. Nature News, 526(7573), 317. 10.1038/526317a 26469026

[ece35020-bib-0073] Tassin, J. , & Kull, C. A. (2015). Facing the broader dimensions of biological invasions. Land Use Policy, 42, 165–169.

[ece35020-bib-0074] The Economics of Ecosystem and Biodiversity (TEEB) (2016). Ecosystem services TEEB, United States. Retrieved from http://www.teebweb.org/resources/ecosystem-services/.

[ece35020-bib-0075] Vilà, M. , Basnou, C. , Pyšek, P. , Josefsson, M. , Genovesi, P. , Gollasch, S. , … Hulme, P. E. (2010). How well do we understand the impacts of alien species on ecosystem services? A pan‐European, cross‐taxa assessment. Frontiers in Ecology and the Environment, 8(3), 135–144.

[ece35020-bib-0076] Vilà, M. , Espinar, J. L. , Hejda, M. , Hulme, P. E. , Jarošík, V. , Maron, J. L. , … Pyšek, P. (2011). Ecological impacts of invasive alien plants: A meta‐analysis of their effects on species, communities and ecosystems. EcologyLetters, 14, 702–708. 10.1111/j.1461-0248.2011.01628.x 21592274

[ece35020-bib-0077] Vilà, M. , & Hulme, P. E. (Eds.) (2017). Impact of biological invasions on ecosystem services Vol. 12 Cham: Springer.

[ece35020-bib-0078] Walther, G.‐R. , Roques, A. , Hulme, P. E. , Sykes, M. T. , Pyšek, P. , Kühn, I. , … Czucz, B. (2009). Alien species in a warmer world: Risks and opportunities. Trends in Ecology & Evolution, 24, 686–693. 19712994 10.1016/j.tree.2009.06.008

[ece35020-bib-0079] Wickham, H. (2009). ggplot2: Elegant graphics for data analysis. New York, NY: Springer‐Verlag.

[ece35020-bib-0080] Withgott, J. , & Brennan, S. R. (2009). Essential environment, (3rd ed.). San Francisco, CA: Pearson Benjamin Cummings.

[ece35020-bib-0081] Wittenberg, R. , & Cock, M. J. (2005). Best practices for the prevention and management of invasive alien species. SCOPE‐Scientific Committee on Problems of the Environment. International Council of Scientific Unions, 63, 209.

[ece35020-bib-0082] Zieritz, A. , Armas, B. , & Aldridge, D. C. (2014). Registry of non‐native species in the Two Seas region countries (Great Britain, France, Belgium and the Netherlands). Neobiota, 23, 65.

