# Supplementary material for: A practical tool for assessing ecosystem services enhancement and degradation associated with invasive alien species
Source: Ecol Evol. 2019 Mar 27;9(7):3918–36. doi: 10.1002/ece3.5020 (PMC6467848; doi:10.1002/ece3.5020)
Supplement: Supplementary file 1 [file ECE3-9-3918-s001.docx]

**Supplementary information**

**Appendix 1.** Correspondence table of the integrated classification and ecosystem services proposed for this study. We integrated three commonly accepted classification schemes from the Millennium Ecosystem Assessment (MA, 2005), the UK National Ecosystem Assessment (UK NEA; Mace et al., 2011), and The Economics of Ecosystem and Biodiversity (TEEB, 2016). The colours indicate the categories of services: provisioning (green), regulating (orange) and cultural (red). N/A indicates that the category was “not available” in a particular classification scheme.

|  | **This study** | **UK NEA** | **MA** | **TEEB** |
| --- | --- | --- | --- | --- |
| **Provisioning** | **Crops or livestock** | Crops, livestock, fish | Food | Food |
|  | **Harvested wild goods** | N/A | Ornamental resources | N/A |
|  |  | N/A | N/A | Medicinal resources |
|  | **Water supply** | Water supply | Fresh water | Fresh water |
|  | **Trees, standing vegetation, peat** | Trees, standing vegetation, peat | Biochemicals | Raw materials |
|  |  |  | Fibre |  |
|  | **Wild species diversity** | Wild species diversity | Genetic resources | N/A |
|  |  | Wild species diversity |  |  |
| **Regulating** | **Detoxification and purification in soils air and water** | Detoxification and purification in soils, air and water | Water purification and waste treatment | Local climate and air quality |
|  |  |  | Air quality regulation | Waste-water treatment |
|  | **Hazard regulation** | Hazard regulation | Natural hazard regulation | Moderation of extreme events |
|  | **Pollination** | Pollination | Pollination | Pollination |
|  | **Disease and pest regulation** | Disease and pest regulation | Pest regulation | Biological control |
|  |  |  | Disease regulation |  |
|  | **Climate regulation** | Climate regulation | Climate regulation | Carbon sequestration and storage |
|  | **Noise regulation** | Noise regulation | N/A | N/A |
|  | **Erosion regulation** | N/A | Erosion regulation | Erosion prevention and maintenance of soil fertility |
| **Cultural** | **Spiritual experience and sense of place** | Environmental settings | Spiritual and religious values | Spiritual experience and sense of place |
|  |  |  | Sense of place |  |
|  | **Aesthetic appreciation and inspiration for culture, art, design** |  | Cultural heritage values | Aesthetic appreciation and inspiration for culture, art, design |
|  |  |  | Cultural diversity |  |
|  |  |  | Aesthetic values |  |
|  |  |  | Inspiration |  |
|  | **Recreation and tourism** |  | Recreation and ecotourism | Recreation and mental and physical health |
|  |  |  |  | Tourism |
|  | **Mental and physical health** |  | Social relations | Recreation and mental and physical health |
|  | **Knowledge systems and educational values** |  | Educational values | N/A |
|  |  |  | Knowledge systems |  |

**Appendix 2. INSEAT first version, used during the piloting phase**. Asterisks indicate where modifications have been made for the final version; the improvements were based on the feedback provided by respondents of the survey and reviewers.

| [*Species name* – Common name]  If you feel that you do not have the knowledge to assess this species, please proceed with the next species.  **Question 1. Spatial occupation**   \| *Score* \| Please describe the *current invasion stage* of this species in [Country/area] \| \| --- \| --- \| \| *1* \| Local – the species is found in a single site or covers a small area of the [country/area], less than 10% of the area of the [country/area] \| \| *2* \| Regional – populations are present in between 10 and 75% of the area of the [country/area] \| \| *3* \| National – populations are present in more than 75% of the area of the [country/area] \| \| *-* \| I do not know \|   **(*)**  **Question 2. Spreading capacity**   \| *Score* \| Please describe the *spreading capacity* of this species \| \| --- \| --- \| \| *1* \| Low potential - the species spreads slowly \| \| *2* \| Medium/moderate potential – the species spreads rapidly but does not double its range in less than 10 years \| \| *3* \| High potential - the species spreads rapidly, doubling its range in less than 10 years \| \| *-* \| I do not know \|   **Question 3. Management effort (**)**   \| *Score* \| Please select the management effort necessary to *eradicate* this species \| \| --- \| --- \| \| *1* \| Unmanageable – management measures cannot control it \| \| *2* \| High – can be controlled with intensive management \| \| *3* \| Medium – can be eradicated with periodic management \| \| *4* \| Low – successfully eradicated with no ongoing management \| \| *-* \| I do not know \|   **Question 4. Ecosystem services impact assessment**  We have designed a semi-quantitative scale that assesses both positive and negative effects of this species. Each score is defined as:   \| *Score* \| Impact score definition (***) \| \| --- \| --- \| \| *4* \| **Intense** positive impact to the ecosystem service; the effect is **non-permanent** \| \| *3* \| **Noticeable** positive impact to the ecosystem service but **short-lasting** \| \| *2* \| Positive impact on the ecosystem service is **too small** to be significant \| \| *1* \| **No impacts** detectable/ecosystem services **not applicable** to this species \| \| *0* \| Negative impact on the ecosystem service is **too small** to be significant \| \| *-1* \| **Noticeable** negative impact to the ecosystem service; damage is **short-lasting** \| \| *-2* \| **Intense** negative impact to the ecosystem service; damage is **non-permanent** \| \| *-3* \| Negative impact to the ecosystem service is both **intense** and **lasting** \| \| *-4* \| **Intense** positive impact to the ecosystem service; the effect is **non-permanent** \| \| *-* \| I don’t know \|   Please score the **general** impact of this species on each ecosystem service and indicate the confidence using the guidance table in Question 1:   \|  \| Score \| \| --- \| --- \| \| **Provisioning services. *These are the products that people obtain from ecosystems.*** \|  \| \| Crops or livestock *(i.e. provision of food)* \|  \| \| Harvested wild goods *(i.e. ornamental/medicinal resources/wild game)* \|  \| \| Trees, standing vegetation, peat *(i.e. fuels and construction material)* \|  \| \| Water supply *(i.e. supply of local water)* \|  \| \| Wild species diversity *(genetic diversity for animal and plant breeding)* \|  \| \| **Regulating services*. These are the benefits obtained from regulation of ecosystem processes.*** \|  \| \| Detoxification and purification in soils, air and water \|  \| \| Climate regulation *(i.e. local (e.g. temperature and precipitation) or global (e.g. carbon sequestration) regulation)* \|  \| \| Hazard regulation *(i.e. moderation of extreme events as floods or storms)* \|  \| \| Pollination \|  \| \| Noise regulation \|  \| \| Erosion regulation *(i.e. Erosion prevention and maintenance of soil fertility)* \|  \| \| **Cultural services. *These are the nonmaterial benefits obtained from ecosystems*** \|  \| \| Spiritual experience and sense of place *(i.e. religious meaning or sense of belonging)* \|  \| \| Aesthetic appreciation and inspiration for culture, art, design \|  \| \| Recreation and tourism \|  \| \| Mental and physical health \|  \| \| Knowledge systems and educational values *(i.e. types of knowledge and basis for education)* \|  \|   **Question 5. One additional question**  Let's suppose that the negative impacts of this species on the environment can be mitigated with an appropriate management of its wild populations.  Do you think this species would then have the **potential** to provide any of the following benefits? Please, choose *yes* for the ecosystem services that can be improved.  [List of ecosystem services]  **Comments (****)**  Please give any comments regarding this assessment or about the potential benefits that this species can provide (e.g. management needed or limitations).  [Comment box] |
| --- | --- | --- | --- | --- | --- | --- | --- | --- | --- | --- | --- | --- | --- | --- | --- | --- | --- | --- | --- | --- | --- | --- | --- | --- | --- | --- | --- | --- | --- | --- | --- | --- | --- | --- | --- | --- | --- | --- | --- | --- | --- | --- | --- | --- | --- | --- | --- | --- | --- | --- | --- | --- | --- | --- | --- | --- | --- | --- | --- | --- | --- | --- | --- | --- | --- | --- | --- | --- | --- | --- | --- | --- | --- | --- | --- | --- | --- | --- | --- | --- | --- | --- | --- | --- | --- | --- | --- | --- | --- | --- | --- | --- | --- | --- |

(*) To determine uncertainty, the revised proforma asks respondents to report the level of confidence in their assessments, and to provide information that support their scores.

(**) Management effort. The scale used to define the management effort was improved, inspired by the work of Booy et al., (2017).

(***) In the revised version, the impact categories are better defined, and use terminologies that avoid making judgements on the effect of IAS on the ES. The new version also includes the option “Data deficient” for acknowledging that the lack of knowledge is because of no existing data or evidence, rather than the unawareness of an individual.

(****) Question 6 was added to gather information that support the expert’s responses.

**Appendix 3. K means clustering.** Best number of clusters and interpretation of the results.

Clustering algorithms find naturally occurring groups in a dataset. The K-means method divides the data points into “k” number of groups in which the sum of squares from these points to the clusters centre is minimized (Hartigan & Wong, 1979). To select the best “k” (number of clusters), the Silhouette Plot method is widely used. A Silhouette Plot is a representation of the cohesion among the points of a cluster and the separation between the points of different clusters (Fig. A3.b) (Rousseeuw, 1987). Using the notation in the Figure, the *silhouette width* (s_i_) is a representation of the suitability of the object to belong to a cluster (j), and the *silhouette of a cluster* is a plot of the of the s_i_ of all the objects (n_j_) of the cluster ranked in decreasing order. The *average silhouette plot width* (S_i_) is the average of the s_i_ of all the clusters and can be used to measure of fitness of the clustering. Values <0.25 indicate that no substantial structure has been found; 0.26-0.50 indicate that the structure is weak and could be artificial; 0.51-0.70 indicate that a reasonable structure has been found, and from 0.71 to 1.0 indicate a strong structure. From all the average silhouette widths of all “k”, the optimum (closest to 1) indicate the “best” number of “k” i.e. the most “naturally” occurring number of clusters.

When interpreting the graph, one should attend both to the *average silhouette plot width* (S_i_) to find the “best” k and the *silhouette width* (s_i_) of each cluster for the validation of consistency: to identify outliers and objects that lie well within their cluster of the ones are merely somewhere in between clusters. For extended information check “Chapter 2. Partitioning Around Medoids (Program PAM)” from “Finding Groups in Data: An Introduction to Cluster Analysis” (Kaufman & Rousseeuw, 2009).

**Figure Appendix 3**. K mean clustering plot and Silhouette plot obtained for the species of the case studies. a) Clustering plot. The 16 case studies are represented in two dimensions and grouped into three clusters. Components 1 and 2 explain 55.03 % of the point variability. b) Silhouette plot, used to select the best number of clusters. From all the possible number of clusters, k=3 has the highest Average Silhouette Width (S_(i)_ = 0.27). This low value indicates weak cohesion among the data points. j: cluster; n_j_: number objects (species) in each cluster; ave_i∈Cj_ s_i_: average width of each cluster.

**Appendix 4. Species selection procedure.**

From the 282 IAS, we selected those that were chosen for factsheets by the GB-Non-Native Species Information Portal (GB-NNSIP). These factsheets contain additional information of well-known, highly damaging species. This way we ensured that species contained in this list were well-studied. The list was reduced to 99 species.

To further reduce the IAS number a bibliographic search was performed in Web of Science. We recorded the number of entries per species with the following search-string: |<"Species"> OR <"Common name">| *AND* |UK OR "United Kingdom" OR GB OR "Great Britain"| *AND* |"non-native" OR alien OR invasive|. Three species of six taxonomical groups with most papers were selected for this study. The final list of IAS included in this study includes 18 species.

The final list of species included in the online assessment is provided below. “No. entries in Web of Science” refers to the number of entries in Web of Science on August 2016; “No. times assessed” refers to the number of times the species was evaluated by the respondents of the survey.

| **Species name** | **Common name** | **No. entries in Web of Science** | **No. times assessed** |
| --- | --- | --- | --- |
| **Higher plants** | | | |
| *Fallopia japonica* | Japanese knotweed | 22 | 28 |
| *Impatiens glandulifera* | Himalayan balsam | 18 | 26 |
| *Rhododendron ponticum* | Rhododendron | 8 | 21 |
| **Insects** | | | |
| *Harmonia axyridis* | Harlequin ladybird | 11 | 12 |
| *Frankliniella occidentalis* | Western flower thrips | 2 | 5 |
| *Leptoglossus occidentalis* | Western conifer seeds bug | 1 | 2 |
| **Aquatic crustaceans** | | | |
| *Pacifastacus leniusculus* | Signal crayfish | 20 | 16 |
| *Dikerogammarus villosus* | Killer shrimp | 15 | 17 |
| *Astacus leptodactylus* | Turkish crayfish | 4 | 12 |
| **Marine plants** | | | |
| *Undaria pinnatifida* | Japanese kelp/Wakame | 4 | 28 |
| *Sargassum muticum* | Japanese wireweed | 3 | 6 |
| *Codium fragile* | Green sea fingers | 1 | 3 |
| **Vertebrates-mammal** | | | |
| *Sciurus carolinensis* | Grey squirrel | 31 | 22 |
| *Mustela vison* | American mink | 24 | 15 |
| *Cervus nippon* | Sika deer | 4 | 10 |
| **Vertebrates-avian** | | | |
| *Psittacula krameri* | Neck-ringed parakeet | 6 | 10 |
| *Branta canadensis* | Canada goose | 3 | 8 |
| *Oxyura jamaicensis* | Ruddy duck | 3 | 10 |
