# Supplementary figures and images for: A practical tool for assessing ecosystem services enhancement and degradation associated with invasive alien species
Source: Ecol Evol. 2019 Mar 27;9(7):3918–36. doi: 10.1002/ece3.5020 (PMC6467848; doi:10.1002/ece3.5020)

a) Clustering plot (k=3)

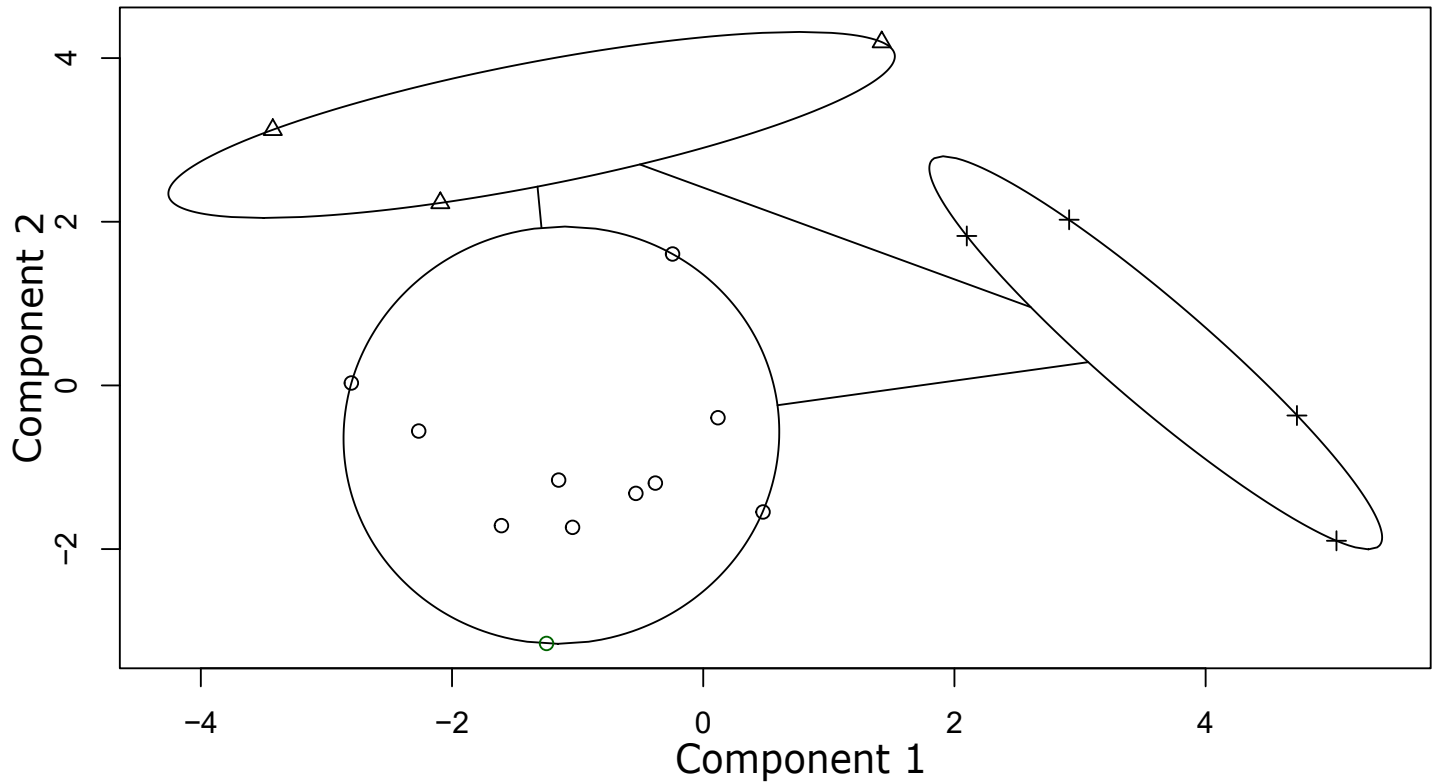

b) Silhouette plot (k=3, n=18)

Average silhouette width ( $S_i$ ) : 0.27

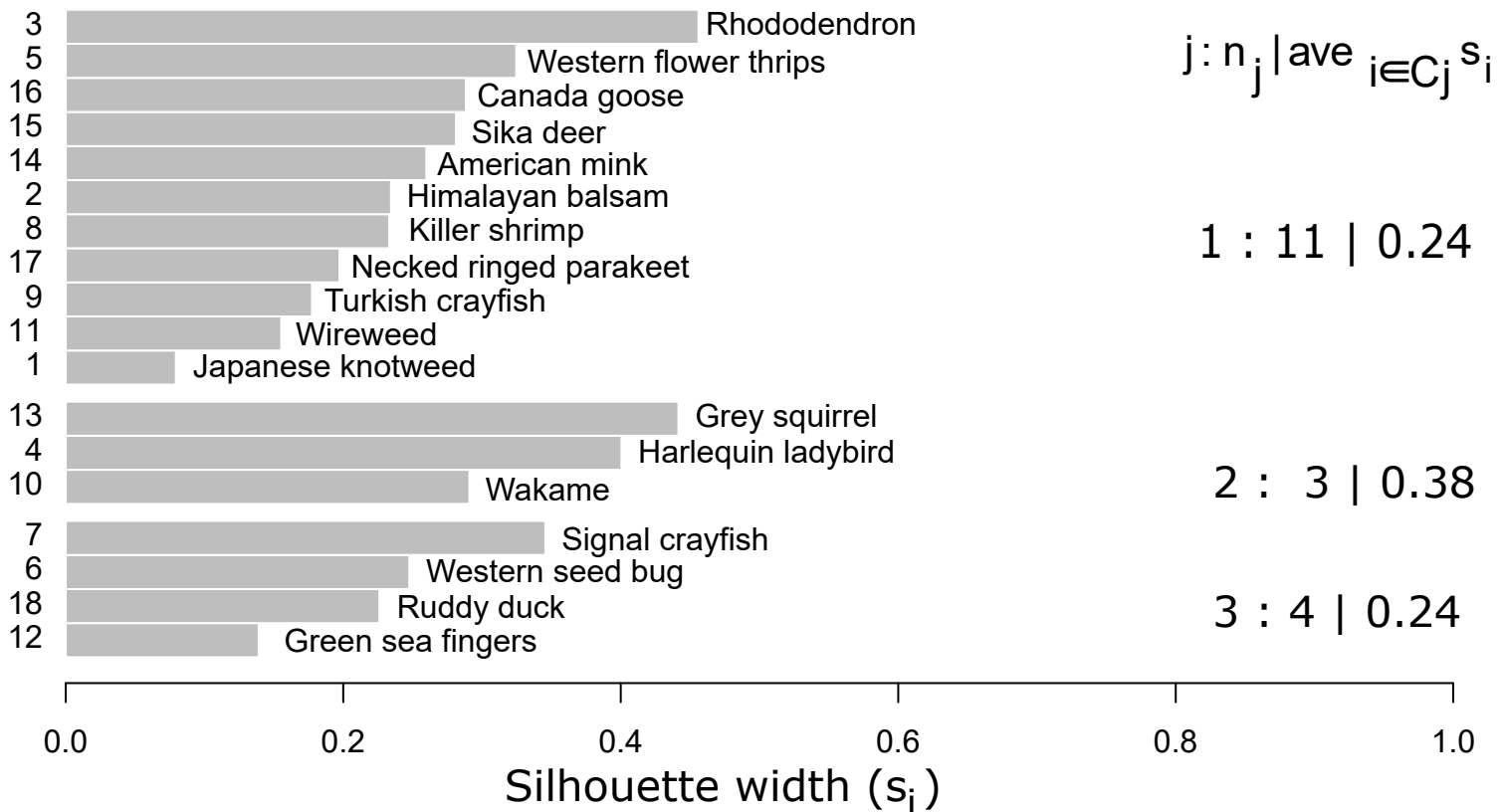

Supplement: Supplementary file 2 [file ECE3-9-3918-s002.pdf]
